# Supplementary material for: Scalable in vivo cardiac functional genomics with compressed AAV-Perturb-seq reveals a common mitochondrial response to perturbation
Source: bioRxiv. 2026 Jun 4:2026.06.01.729445. Preprint. [Version 1] doi: 10.64898/2026.06.01.729445 (PMC13252024; doi:10.64898/2026.06.01.729445)
Supplement: Supplement 5 [file NIHPP2026.06.01.729445v1-supplement-5.pdf]

bioRxiv preprint doi: https://doi.org/10.64898/2026.06.01.729445; this version posted June 5, 2026. The copyright holder for this preprint (which was not certified by peer review) is the author/funder, who has granted bioRxiv a license to display the preprint in perpetuity. It is made available under a CC-BY 4.0 International license.

642 **SUPPLEMENTARY FIGURE TITLES AND LEGENDS:**

bioRxiv preprint doi: https://doi.org/10.64898/2026.06.01.729445; this version posted June 5, 2026. The copyright holder for this preprint (which was not certified by peer review) is the author/funder, who has granted bioRxiv a license to display the preprint in perpetuity. It is made available under a CC-BY 4.0 International license.

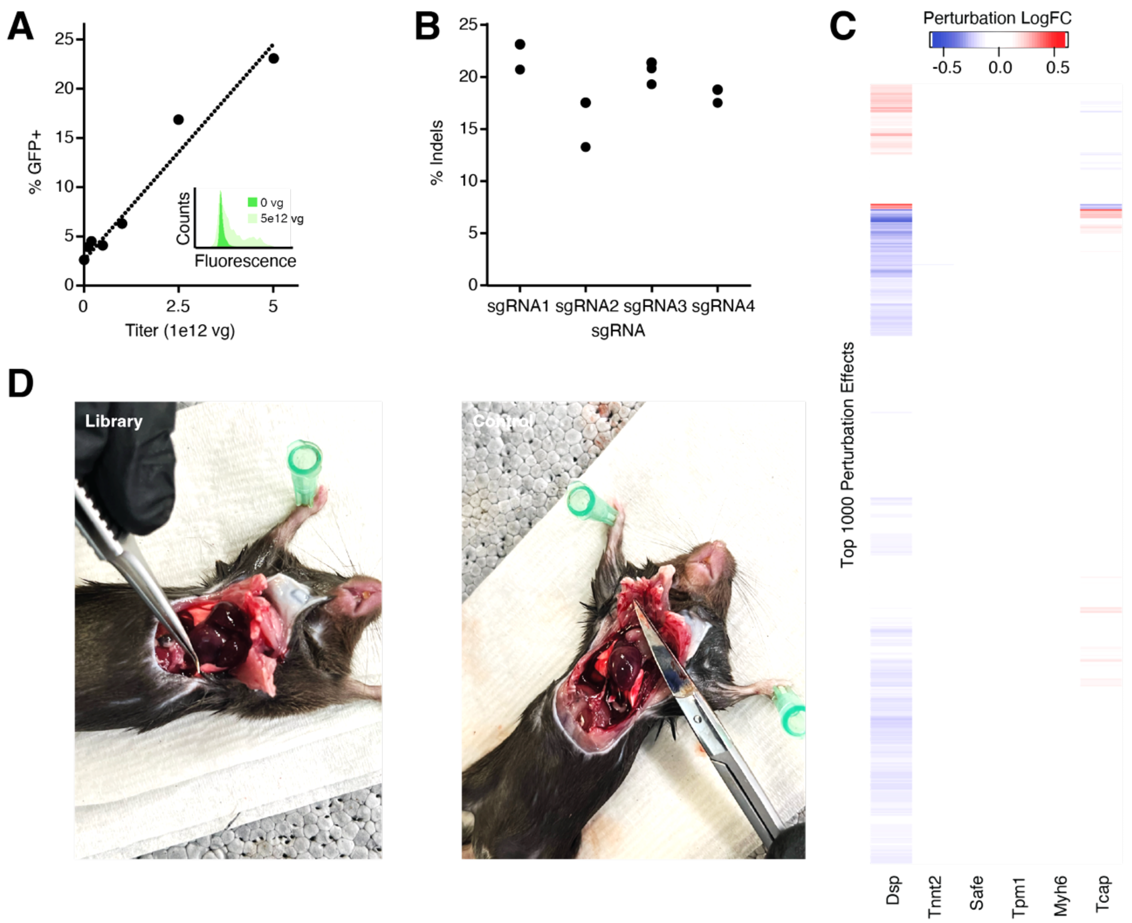

Figure S1. MyoAAV-mediated delivery allows for cardiac in vivo Perturb-seq, related to Figure 1. (A) In vivo titration of MyoAAV cardiac transduction efficiency shows a near linear relationship between viral titer and nuclear GFP positivity. **(B)** MyoAAV-mediated delivery of Pank1 sgRNA allows for efficient indel generation in the murine heart. **(C)** Heatmap of top 1000 perturbation effects after delivery of sgRNA for the corresponding gene. Insignificant effects ($q < 0.2$) are shaded white. **(D)** Delivery of sgRNA library at high MOI leads to cardiac chamber dilation.

bioRxiv preprint doi: https://doi.org/10.64898/2026.06.01.729445; this version posted June 5, 2026. The copyright holder for this preprint (which was not certified by peer review) is the author/funder, who has granted bioRxiv a license to display the preprint in perpetuity. It is made available under a CC-BY 4.0 International license.

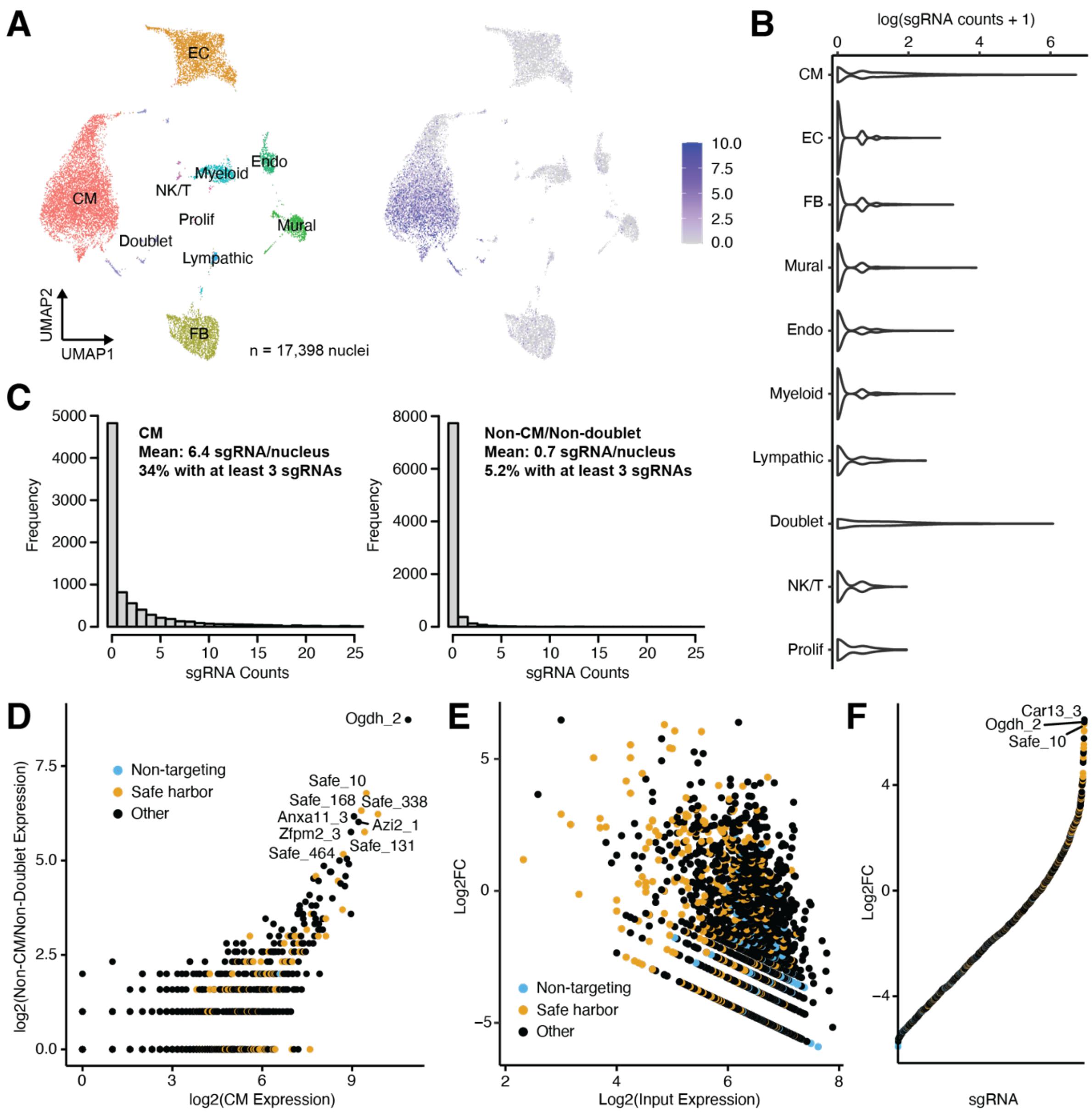

651

bioRxiv preprint doi: https://doi.org/10.64898/2026.06.01.729445; this version posted June 5, 2026. The copyright holder for this preprint (which was not certified by peer review) is the author/funder, who has granted bioRxiv a license to display the preprint in perpetuity. It is made available under a CC-BY 4.0 International license.

Figure S2. Technical characteristics of Perturb-seq screen, related to Figure 2. (A) UMAP showing isolated cell type identity (left) and colored by sgRNA count (right). **(B)** The majority of sgRNAs were assigned to cardiomyocyte or doublet nuclei. **(C)** Cardiomyocyte nuclei contained a mean of 6.4 sgRNA per nuclei, with ~34% having at least 3 sgRNA (left). Non-cardiomyocyte/non-doublet nuclei contained a mean of 0.7 sgRNA per nuclei, with ~5.2% having at least 3 sgRNA (right). **(D)** sgRNA assignment to non-cardiomyocytes correlated with the abundance of the same sgRNA in cardiomyocytes. **(E)** Observed fold-change in sgRNA abundance in cardiomyocytes versus in input library as a function of abundance in the input library. Note the trend for non-targeting guides to be poorly recovered from cardiomyocyte nuclei. **(F)** Ranked observed fold-change in sgRNA abundance in cardiomyocytes versus in input library.

bioRxiv preprint doi: https://doi.org/10.64898/2026.06.01.729445; this version posted June 5, 2026. The copyright holder for this preprint (which was not certified by peer review) is the author/funder, who has granted bioRxiv a license to display the preprint in perpetuity. It is made available under a CC-BY 4.0 International license.

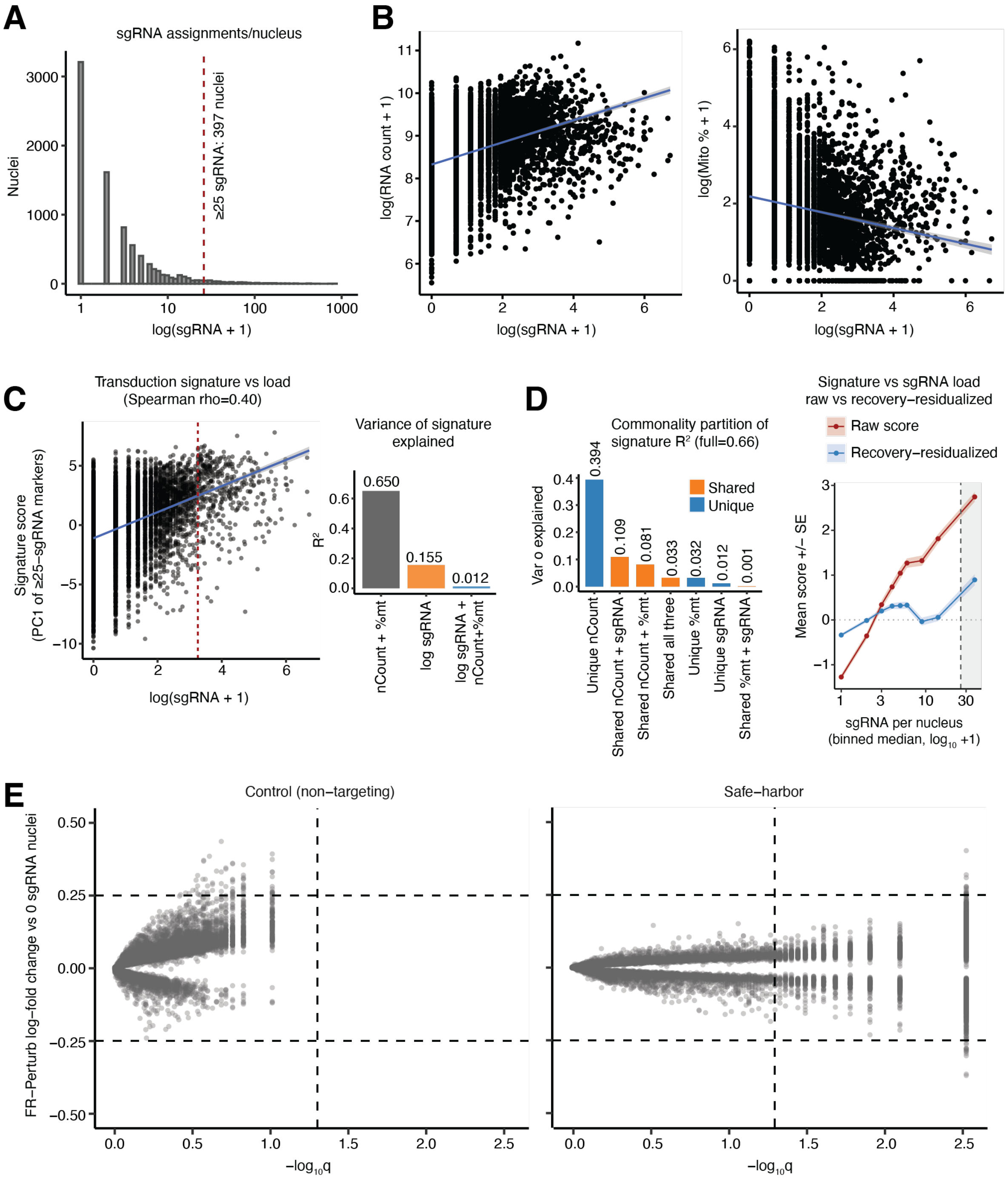

bioRxiv preprint doi: https://doi.org/10.64898/2026.06.01.729445; this version posted June 5, 2026. The copyright holder for this preprint (which was not certified by peer review) is the author/funder, who has granted bioRxiv a license to display the preprint in perpetuity. It is made available under a CC-BY 4.0 International license.

Figure S3. Apparent transduction-load transcriptional signature is overwhelmingly technical, and perturbation-effect estimation is robust to the control/normalization choice, related to Figure 2. (A) Distribution of assigned sgRNAs per cardiomyocyte nucleus ($\log_{10}$ scale); dashed line marks the ≥25-sgRNA "super-transducer" threshold (max 828 sgRNAs per nucleus). **(B)** Across cardiomyocyte nuclei, apparent sgRNA load is positively coupled to transcript recovery and inversely to mitochondrial content (Spearman $\rho = +0.38$ vs log nCount; $\rho = -0.23$ vs log %mito), i.e. guide and transcript capture covary. **(C)** A transduction-signature score (first principal component of the 406 genes upregulated in ≥25-sgRNA cardiomyocyte nuclei; **left**) is largely technical: total UMI count and mitochondrial fraction explain $R^2 = 0.65$ of its variance, whereas log sgRNA load adds only +1.2% beyond them (added $R^2 = 0.012$; **right**). **(D)** Commonality (variance) partition of the signature score (regressed on nCount, %mito and log sgRNA; R^2_full = 0.66; **left**). Of sgRNA load's gross association ($R^2 \approx 0.155$) only 0.012 is unique to sgRNA whereas 0.109 is shared with recovery (nCount), i.e. its apparent effect is overwhelmingly collinear with transcript recovery. Mean signature score across binned sgRNA load, raw versus recovery-residualized (residuals of score ~ nCount + %mito; **right**). Linear recovery adjustment removes ~70–80% of the apparent dose-response (raw range ≈ 4.0 → residual ≈ 1.2; the residualized curve is flat across most of the sgRNA transduction range. The residual signal that remains is confined to the shaded marker-defining ≥25-sgRNA bin, where the relationship is circular (the 406 markers were defined from those nuclei). **(E)** FR-Perturb effects of the non-targeting "Control" and "Safe-harbor" perturbation columns (vs the 0 sgRNA reference) are near-null (0 and 30 genes at $|LFC| > 0.25$ and $q < 0.05$; median $|LFC| \approx 0.04$), demonstrating that estimated perturbation effects are not an artifact of the control/normalization choice.

bioRxiv preprint doi: https://doi.org/10.64898/2026.06.01.729445; this version posted June 5, 2026. The copyright holder for this preprint (which was not certified by peer review) is the author/funder, who has granted bioRxiv a license to display the preprint in perpetuity. It is made available under a CC-BY 4.0 International license.

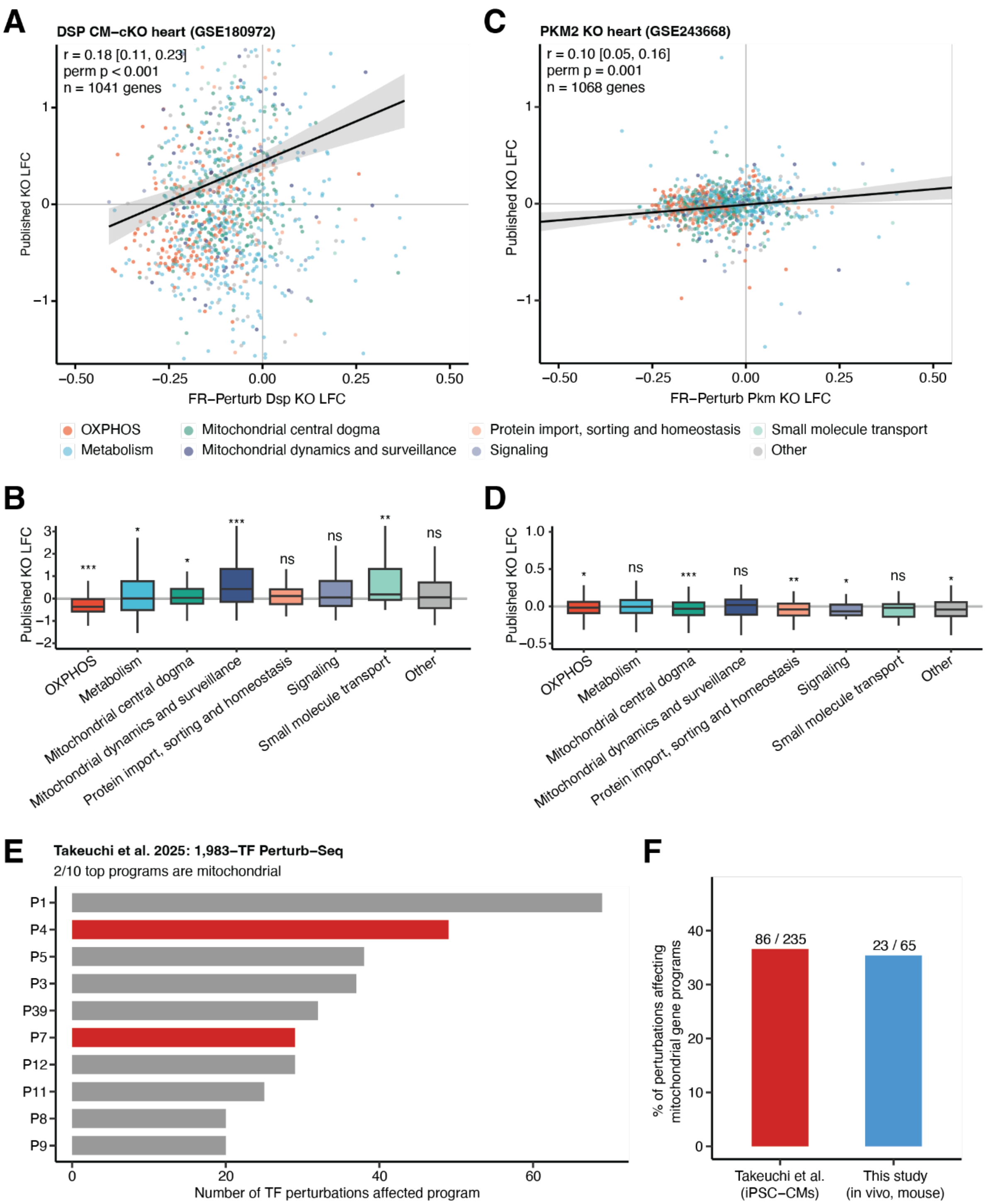

688

bioRxiv preprint doi: https://doi.org/10.64898/2026.06.01.729445; this version posted June 5, 2026. The copyright holder for this preprint (which was not certified by peer review) is the author/funder, who has granted bioRxiv a license to display the preprint in perpetuity. It is made available under a CC-BY 4.0 International license.

Figure S4. Mitochondrial transcriptome dysregulation is a conserved response to cardiac perturbation across independent screens, related to Figure 3. (A) Correlation between FR-Perturb log-fold changes (this study) and published bulk RNA-seq log-fold changes from DSP cardiomyocyte-specific conditional KO hearts (GSE180972). Each point represents one MitoCarta 3.0 gene, colored by mitochondrial functional category. Pearson r with bootstrap 95% CI and permutation p-value are shown. LFCs estimated using DESeq2. **(B)** Distribution of published DSP KO log-fold changes across MitoCarta 3.0 functional categories. Boxes show median ± IQR; asterisks indicate Wilcoxon signed-rank test against 0 (p < 0.05, ** p < 0.01, *** p < 0.001; minimum 5 genes per category). **(C-D)** As in (A-B) for PKM2 cardiac KO mice (GSE243668; PKM2$^{fl/fl}$ versus KO at baseline). **(E)** The 10 gene programs most commonly disrupted across 1,983 TF perturbations in the Takeuchi et al. 2025 iPSC-cardiomyocyte Perturb-seq dataset, ranked by the number of TF knockdowns affecting each program. Programs with ≥10% overlap with MitoCarta 3.0 genes (top 300 program members) are highlighted in red. **(F)** Fraction of TF perturbations affecting at least one mitochondrial gene program in the Takeuchi et al. 2025 iPSC-CM screen (human, *in vitro*) versus this study (mouse, *in vivo*). Mitochondrial programs defined as described in (E); perturbations in this study classified using FR-Perturb q < 0.2.

bioRxiv preprint doi: https://doi.org/10.64898/2026.06.01.729445; this version posted June 5, 2026. The copyright holder for this preprint (which was not certified by peer review) is the author/funder, who has granted bioRxiv a license to display the preprint in perpetuity. It is made available under a CC-BY 4.0 International license.

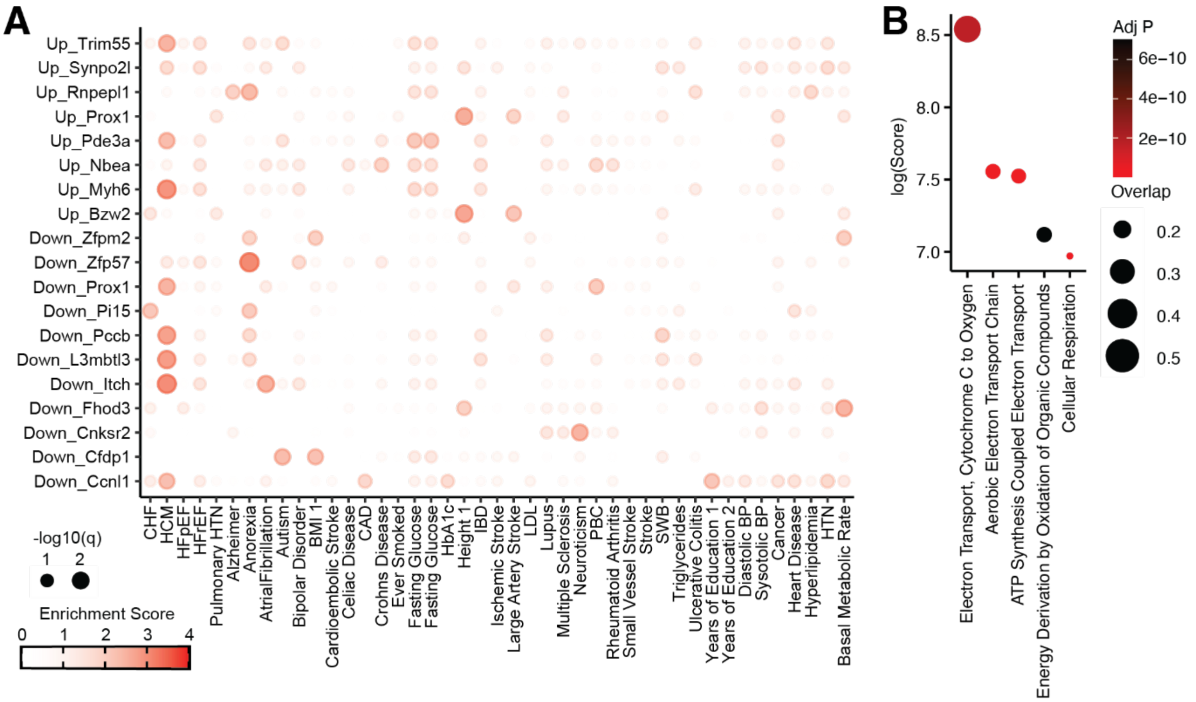

707

bioRxiv preprint doi: https://doi.org/10.64898/2026.06.01.729445; this version posted June 5, 2026. The copyright holder for this preprint (which was not certified by peer review) is the author/funder, who has granted bioRxiv a license to display the preprint in perpetuity. It is made available under a CC-BY 4.0 International license.

Figure S5. Integration of Perturb-seq with GWAS highlights the role of mitochondrial dysfunction in hypertrophic cardiomyopathy, related to Figure 3. (A) Sc-linker computed heritability enrichment scores of perturbation signatures (rows) across human traits (columns). Signatures are defined by a weighted collection of altered downstream genes for each perturbation. “Up” indicates that the corresponding signature is the set of genes that are upregulated by perturbation while “down” represents the opposite. Only perturbations/traits with at least one entry with $q < 0.5$ are shown. **(B)** Gene ontology enrichment of the union of the top 50 heaviest weighted downstream genes making up the signatures of perturbations with $q < 0.2$ for hypertrophy cardiomyopathy.

bioRxiv preprint doi: https://doi.org/10.64898/2026.06.01.729445; this version posted June 5, 2026. The copyright holder for this preprint (which was not certified by peer review) is the author/funder, who has granted bioRxiv a license to display the preprint in perpetuity. It is made available under a CC-BY 4.0 International license.

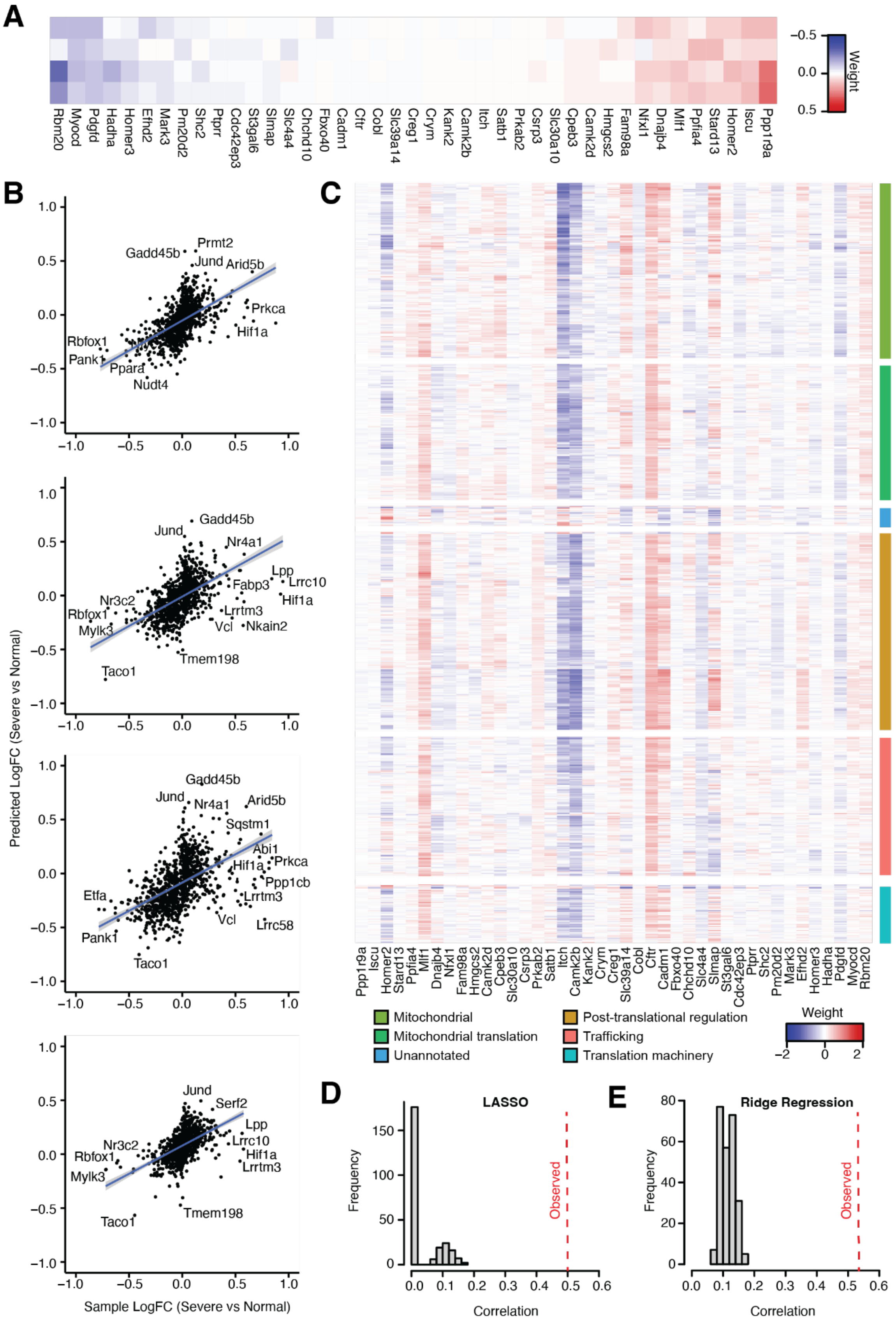

bioRxiv preprint doi: https://doi.org/10.64898/2026.06.01.729445; this version posted June 5, 2026. The copyright holder for this preprint (which was not certified by peer review) is the author/funder, who has granted bioRxiv a license to display the preprint in perpetuity. It is made available under a CC-BY 4.0 International license.

Figure S6. Reconstruction of the murine RVF transcriptome using gene perturbation signatures, related to Figure 4. (A) Heatmap showing weights of LASSO (alpha = 1) minimized perturbation-set which replicates the transcriptional signature of murine RVF. **(B)** Dot plots showing measured versus fit fold changes between healthy and failing murine RVs across 4 samples. **(C)** Heatmap showing effects of selected perturbations on downstream genes. The observed fit is significantly better, as measured by linear correlation between predicted vs measured fold change, than what would be observed by random chance, as calculated by randomly permuting downstream genes and re-fitting data with either LASSO **(D)** or ridge regression **(E)**.

bioRxiv preprint doi: https://doi.org/10.64898/2026.06.01.729445; this version posted June 5, 2026. The copyright holder for this preprint (which was not certified by peer review) is the author/funder, who has granted bioRxiv a license to display the preprint in perpetuity. It is made available under a CC-BY 4.0 International license.

SUPPLEMENTARY DATA TITLES AND LEGENDS:

Data S1. Pilot sgRNA library, related to Figure 1.

Data S2. Full screen sgRNA library (585 genes), related to Figure 2.

Data S3. Gene modules, related to Figure 2.

Data S4. Gene Ontology modules, related to Figure 4.
